# pRRophetic: An R Package for Prediction of Clinical Chemotherapeutic Response from Tumor Gene Expression Levels

**DOI:** 10.1371/journal.pone.0107468

**Published:** 2014-09-17

**Authors:** Paul Geeleher, Nancy Cox, R. Stephanie Huang

**Affiliations:** 1 Section of Hematology/Oncology, Department of Medicine, University of Chicago, Chicago, Illinois, United States of America; 2 Section of Genetic Medicine, Department of Medicine, University of Chicago, Chicago, Illinois, United States of America; University of Hawaii Manoa, United States of America

## Abstract

We recently described a methodology that reliably predicted chemotherapeutic response in multiple independent clinical trials. The method worked by building statistical models from gene expression and drug sensitivity data in a very large panel of cancer cell lines, then applying these models to gene expression data from primary tumor biopsies. Here, to facilitate the development and adoption of this methodology we have created an R package called *pRRophetic*. This also extends the previously described pipeline, allowing prediction of clinical drug response for many cancer drugs in a user-friendly R environment. We have developed several other important use cases; as an example, we have shown that prediction of bortezomib sensitivity in multiple myeloma may be improved by training models on a large set of neoplastic hematological cell lines. We have also shown that the package facilitates model development and prediction using several different classes of data.

## Introduction

Robust prediction of *in vivo* chemotherapeutic response, using before treatment (baseline) gene expression and drug sensitivity data gathered on cancer cell lines, has been a long standing and controversial problem in pharmacogenomics. We recently presented a solution to this problem [1]. Our method fitted models for baseline gene expression against drug sensitivity in a very large panel of cell lines; following data homogenization and filtering, these models were applied to baseline expression levels from primary tumor biopsies, yielding *in vivo* drug sensitivity predictions. We showed that this approach captured variability in clinical response in multiple independent clinical trials and obtained predictions approximately as good as, or better than, gene signatures derived directly from clinical data. The method involved the integration of several sophisticated analytical and statistical tools. Here, we present an R package that implements this pipeline as a small number of easy-to-use functions. Furthermore this tool allows prediction of non-clinical phenotypes, user-defined training sets and facilitates prediction of both continuous and categorical phenotypes. Prediction accuracy can be estimated using k-fold or leave-one-out cross-validation. There are built-in functions for interpreting results and we have also provided extensive documentation.

## Methods

A complete technical description of the prediction pipeline implemented in the *pRRophetic* package is described in [1]. Briefly, microarray probes are (when possible) first remapped to the latest build of EntrezGene. Training and test expression data are quantile normalized separately and subsequently combined by standardizing the mean and variance of each gene using an empirical Bayesian approach. Genes with very low variability across samples are removed. A ridge regression model is fit to the training expression data using all remaining genes as predictors and the drug sensitivity (IC_50_) values (of the drug of interest) as the outcome variable. Finally, this model is applied to the processed, standardized, filtered clinical tumor expression data, yielding a drug sensitivity estimate for each patient. All R source code is publicly available via GitHub and on our website (see “Availability” section).

## Results and Discussion

### Primary use case

The *pRRophetic* package can be used for phenotype prediction from gene expression microarray data. The novel use case is prediction of clinical chemotherapeutic response using only baseline tumor gene expression data. This is achieved by creating statistical models from the gene expression and drug sensitivity data from cell lines in the Cancer Genome Project (CGP) [2]; these models are then applied to tumor gene expression levels in primary tumor samples, yielding an *in vivo* drug sensitivity prediction. In addition, we show that the package can be used to accurately predict drug sensitivity in an additional panel of cell lines and that clinical datasets can also be used for model development.

### Predicting clinical chemotherapeutic response

**Figure 1:**
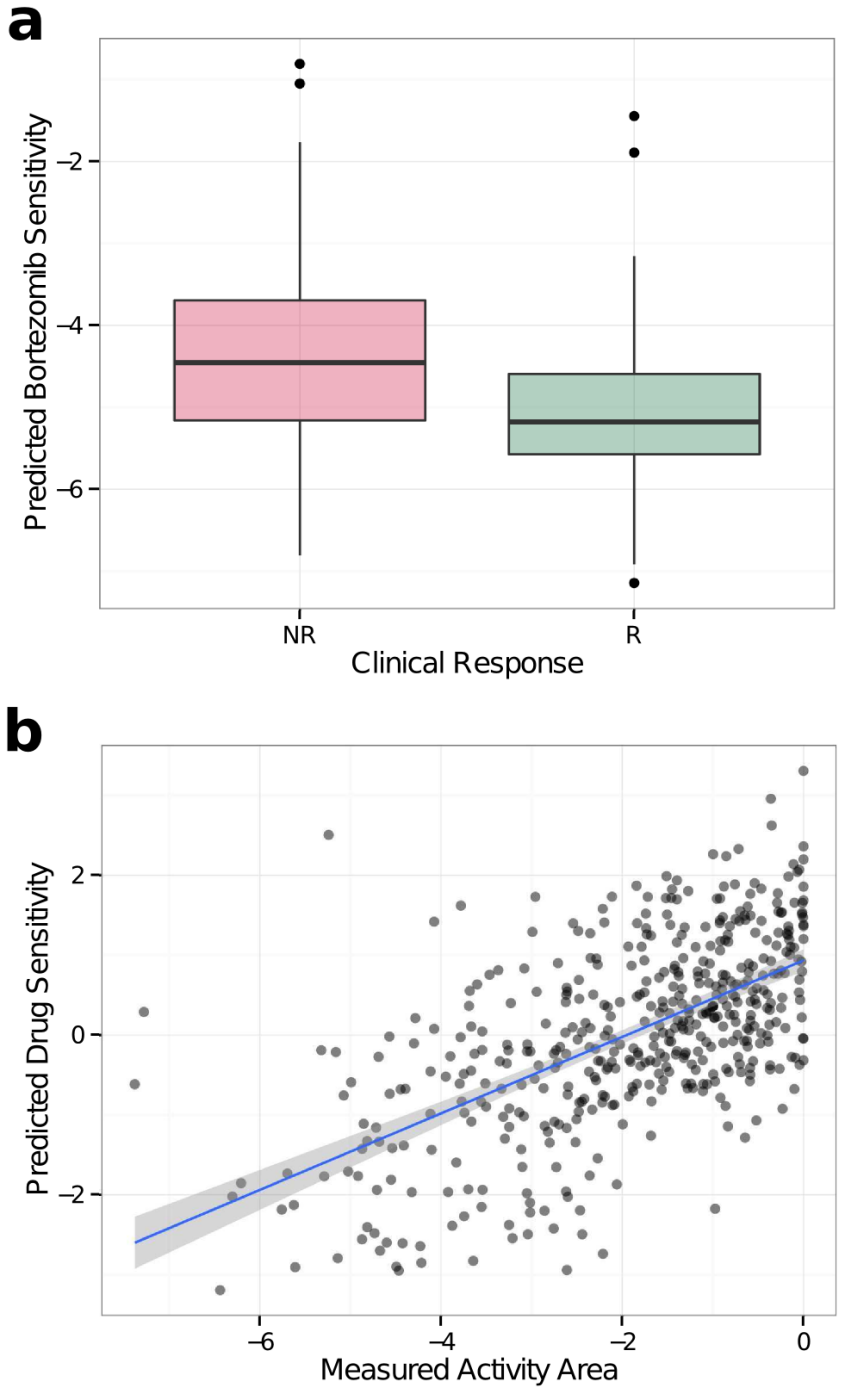
The prediction accuracy achieved in external datasets. (a) A boxplot showing the predicted clinical bortezomib sensitivity for multiple myeloma patients. The predictions where made using the *pRRopheticPredict()* function for only hematological cancer cell lines. (NR, Clinical Non-responders; R, Clinical Responders) (b) The predicted PD0325901 sensitivity in CCLE, plotted against the measured activity area (a measure of drug response) in CCLE. A linear regression line and 95% confidence intervals are included.

We previously reported that this pipeline (using models developed on all available CGP cell lines) could enrich for responders to the proteosome inhibitor bortezomib in multiple myeloma [1]. Using the *pRRophetic* package, this result can be achieved using a single line of R code, once the clinical gene expression data are correctly arranged. Here, we have implemented functionality that allows the user to specify different classes of cell lines from which to perform prediction. By adjusting this parameter, the prediction of bortezomib sensitivity in myeloma can be improved by training the models using only cell lines derived from hematological cancers. Clinical trial-defined “sensitive” and “resistant” patients were separated with *P* = 2.9×10^−5^ (from t-test; Fig. 1(a); for Affymetrix U133A arrays) compared to *P* = 1.5×10^−3^ when using models derived from all CGP cell lines. Interestingly, models derived from only solid tumor cell lines do not significantly separate these groups, suggesting that considering biological context may improve model development. This result is strongly consistent with expectation and provides further supports for the validity of the approach. These results can be easily generated using the *pRRopheticPredict()* function. Clinically, it is common practice to report dichotomous predictions, for example classifying patients as either “sensitive” or “resistant” to a drug. Hence, we have included functions that estimate a cutpoint using the mean IC_50_ value in the training data, thus segmenting patients into two groups based on their predicted drug sensitivity. Using this cutpoint, clinical terms (that typically apply to classifiers) such as positive predictive values (PPV) and negative predictive values (NPV) can be calculated. The models above achieved PPV of 57% or 63% and NPV of 59% or 69% when derived from all tissues or only blood, respectively. Similar use cases are fully detailed in the package vignette.

### Predicting *in vitro* drug sensitivity

Another use case is drug sensitivity prediction in additional panels of cell lines (e.g. where response to a particular drug has not yet been measured). Thus, as a proof-of-concept we applied CGP derived models (using the *pRRopheticPredict()* function) to data from the Cancer Cell Line Encyclopedia (CCLE) [3] for the MEK inhibitor PD0325901.The predicted and measured drug sensitivity values were highly correlated (Fig. 1(b); Spearman correlation (*r_s_*)  = 0.58, *P*<2.2×10^−16^). Encouragingly, this result is consistent with 5-fold cross-validation on the training set (performed using the *pRRopheticCV()* function; *r_s_* = 0.57, *P*<2.2×10^−16^). These represent similar correlations to those observed when drug sensitivity was compared for cell lines that overlap both CGP and CCLE [4]. This suggests that *in vitro* sensitivity to PD0325901 can be predicted very accurately from gene expression data. Future investigations may focus on whether a similarly impressive accuracy can be achieved in clinical data.

### Prediction by training on a clinical dataset

For model development, the training data is not limited to CGP. In fact, using the *calcPhenotype()* or *classifySamples()* functions (for linear and logistic models respectively), a user can specify their own training dataset; for example, data generated on a new panel of cell lines, or even clinical data. In theory, any relevant system could be used for learning. As a proof-of concept we used *pRRophetic* to build models on two arms (025 and 040) of the bortezomib clinical trial (discussed above; as per [5]) and predicted drug sensitivity on the remaining arm (039). Using this approach, the predicted drug sensitivity for trial defined “sensitive” and “resistant” patients was significantly different (*P* = 0.02 from t-test), suggesting that some signal is being captured and that this is a viable application of this tool. However, this underperformed when compared to the CGP derived models, potentially due to the greater number of samples, a more precisely measured drug response in CGP or possible confounding factors in the clinical dataset.

## Conclusions

We have developed an R package that can predict phenotypes from gene expression microarray data. The primary use case is predicting clinical chemotherapeutic response using the CGP cell lines as a training set, but it can also be applied to non-clinical data and leverage other classes of data (for example clinical data) for model development. We emphasize that the accuracy of clinical predictions has thus only been tested for drugs for which appropriate clinical datasets are available (as discussed in [1]). While this package can generate a prediction for any of the drugs in CGP, the accuracy will ultimately vary from drug to drug based on the predictive power of expression under that particular set of conditions and the appropriateness of cell lines as a model of *in vivo* drug response. It should not be assumed that the prediction accuracy for untested drugs will be the same as for those which have already been tested; however, in addition to the functionality described here, this package will facilitate the testing of such an approach on clinical data that become available. Overall, this work represents a tentative first step towards the ideal of data driven clinical decision making, whereby the best cancer treatment regimens are individualized based on statistical models derived directly from hard data. While this may still be some time from becoming a reality in clinic, we have presented a first step to such an infrastructure that can become the basis for future research in this area.

## Availability

The R package can be downloaded from our website (http://genemed.uchicago.edu/~pgeeleher/pRRophetic) or GitHub (https://github.com/paulgeeleher/pRRophetic).
